# American Resorts, with Notes upon Their Climate

**Published:** 1889-06

**Authors:** 


**American Resorts, with Notes upon their Climate.** By Bushrod W. James, A. M., M. D., Member of the American Association for the Advancement of Science; the American Public Health Association; the Pennsylvania Historical Society; the Franklin Institute, and the Academy of Natural Sciences, Philadelphia; the Society of Alaskan Natural History and Ethnology, Sitka, Alaska; etc., etc. With a translation from the German by Mr. S. Kauffmann of those chapters of "Die Klimate der Erde," written by Dr. A. Woeikof, of St. Petersburg, Russia, that relate to North and South America and the islands and oceans contiguous thereto. Intended for invalids and those who desire to preserve good health in a suitable climate. Copyrighted. Philadelphia and London: F. A. Davis. 1889. Philadelphia, U. S. A., 1231 Filbert street; London, Eng., 139-143 Oxford street W. Octavo, 300 pages, cloth. Price, $2.00.

The evident design of the author in the preparation of this work has been to popularize American health stations, and to make Americans more familiar with the numerous and valuable resorts in our own land. He has brought together in a single, neat, and well-printed volume a vast amount of useful information that deserves to be well studied by both profession and laity.

In the chapter on Medical Climatology—the first in the book—Dr. James displays a knowledge of his subject which wins the favor of his professional audience at once; and in the second, on Benefits and Dangers of Health Resorts, he gives most wholesome advice, clothed in such pleasant language that it makes delightful reading. His description of the many resorts, classified as to their various geographical location, or special health-giving qualities, and again as to seasons, is a most attractive feature of the book, and may be examined with interest by those in search of both health and pleasure.

The paragraph devoted to Lake Chautauqua contains a piece of information that will surprise our Jamestown neighbors when it informs them that their enterprising city, located at the southern end or outlet, "is a town of *six hundred* inhabitants;" but it may somewhat appease their ire to learn, in the next sentence, that this is "a popular place, with good hotels and boarding places."

Buffalo is described as "situated on the foot of the lake (Erie), at the mouth of the Buffalo river, and at the head of the Niagara river, about thirty-two miles from the falls; this is a prosperous place of considerable commercial importance (whew!) and has a population of about two hundred and forty thousand." A city with a quarter million inhabitants, the greatest railway center on the continent, and with unrestricted use of the water navigation of the great lakes and the Erie canal may, with a truth, be said to have *some* commercial importance.

The chapter on Therapeutics considers intelligently the application of the principles of climate to such diseases as, in the main, may be reasonably expected to derive benefit therefrom, and will repay careful reading.

Mr. Kauffmann's translation of Dr. Woeikof's "Die Klimate der Erde" serves as a most fitting conclusion to this instructive book. Here one may obtain valuable information regarding the High North, the Middle Latitudes of North America, Tropical America, South America, and the Atlantic Ocean. A copious index serves the great convenience of the reader. Taken altogether, this is by far the most complete exposition of the subject of resorts that has yet been put forth, and it is one that every physician must needs possess intelligent information upon. We predict a large demand for this useful and attractive book.

